# Association between Clinical Use of Colchicine and Risk of Type 2 Diabetes Mellitus among Gouty Patients: A Nationwide Cohort Study

**DOI:** 10.3390/ijerph19063395

**Published:** 2022-03-14

**Authors:** Chen-Chih Chu, Yong-Chen Chen, Ming-Hsun Lin, Wen-Tung Wu, Feng-Cheng Liu, Hsiang-Cheng Chen, Yu-Ching Chou, Chien-An Sun

**Affiliations:** 1 Division of Rheumatology/Immunology and Allergy, Department of Internal Medicine, Tri-Service General Hospital, National Defense Medical Center, Taipei 114, Taiwan; john30321@hotmail.com (C.-C.C.); lfc10399@yahoo.com.tw (F.-C.L.); alex0624x@gmail.com (H.-C.C.); 2 Data Science Center, College of Medicine, Fu-Jen Catholic University, New Taipei City 242, Taiwan; yongchenchen0824@gmail.com; 3 Department of Medicine, College of Medicine, Fu-Jen Catholic University, New Taipei City 242, Taiwan; 4 Division of Endocrinology and Metabolism, Department of Internal Medicine, Tri-Service General Hospital, National Defense Medical Center, Taipei 114, Taiwan; tim6801@msn.com; 5 Department of Pharmacy, Tri-Service General Hospital, National Defense Medical Center, Taipei 114, Taiwan; allen541312@gmail.com; 6 School of Public Health, National Defense Medical Center, Taipei 114, Taiwan; trishow@mail.ndmctsgh.edu.tw; 7 Department of Public Health, College of Medicine, Fu-Jen Catholic University, No. 510, Zhongzheng Road, Xinzhuang District, New Taipei City 24205, Taiwan

**Keywords:** cohort study, colchicine, diabetes mellitus, gout

## Abstract

Background: Gout is the most common form of inflammatory arthritis in adults. Even though a link between gouty arthritis and type 2 diabetes mellitus (T2DM) has been reported, there is a limited understanding of the association between the anti-inflammatory agent colchicine and the risk of T2DM. This aim of this study was to assess the association between the use of colchicine and the risk of T2DM in an Asian cohort. Methods: A retrospective cohort study was conducted using the National Health Insurance Research Database (NHIRD) in Taiwan from 2000 to 2013. The study cohorts comprised 3841 gouty patients using colchicine (the exposed cohort) and 7682 gouty patients not using colchicine (the unexposed -cohort). The primary outcome was incident DM. The hazard ratios (HRs) and 95% confidence intervals (CIs) derived from a Cox proportional regression model were used to assess the association between colchicine use and the risk of diabetes. Results: The cumulative incidence of T2DM was significantly lower in the exposed cohort (18.8%) than in the unexposed cohort (25.0%). The risk of T2DM was significantly lower in colchicine users than in non-users (adjusted HR, 0.74; 95% CI, 0.36–0.87). The inverse relationship between colchicine use and diabetes risk remained consistent across sex and age groups. Conclusions: This cohort study provides longitudinal evidence that the use of colchicine is associated with a reduced risk of T2DM. This conclusion, however, needs to be interpreted cautiously given the lack of body mass index data in the NHIRD. Further studies are needed to determine the clinical implications of this study.

## 1. Introduction

Gout is the most common form of inflammatory arthritis, which is caused by the deposition of monosodium urate crystals within joints after chronic hyperuricemia [1]. It has been noted that gout and hyperuricemia are associated with type 2 diabetes mellitus (T2DM) [2,3], metabolic syndrome [4], cardiovascular diseases [5,6], and hypertension [7]. In a clinical setting, the treatment of pain and inflammation among gouty patients is based on non-steroidal anti-inflammatory drugs, corticosteroids, or colchicine. Colchicine is an anti-inflammatory agent and has been involved in the management of patients with gout for centuries [8].

T2DM is a lifestyle disease and is associated with ageing, obesity, unhealthy eating habits, and low levels of physical activity [9]. Clinical trials using IL-1 antagonists to directly target pro-inflammatory factors in patients with T2DM support a potential role for inflammation in the pathogenesis of this condition [10,11]. Therapeutic strategies aimed at anti-inflammation may beneficially influence the course of T2DM. Colchicine is an anti-inflammatory drug with hypoglycemic effects [8,12] and may reduce the risk of T2DM [13]. However, a clear link between colchicine use and the risk of T2DM has yet to be confirmed. In this nationwide retrospective cohort study, the association between colchicine exposure and the risk of T2DM was assessed using a well-established, population-based dataset from Taiwan’s National Health Insurance Research Database (NHIRD).

## 2. Materials and Methods

### 2.1. Data Source

The current study is a population-based retrospective cohort study using a medical claims dataset from the NHIRD. The NHIRD contains comprehensive health care information, including demographic data of insured individuals and data of clinical visits, diagnostic codes, and prescription details, as described previously [14]. Patients’ diagnoses in the database were encoded using the International Classification of Diseases, Ninth revision, Clinical Modification (ICD-9-CM). The NHIRD has been used for high-quality epidemiological studies with good data validity regarding diagnoses, drug prescriptions, and hospitalizations [15,16,17,18]. The data of this study were obtained from the Longitudinal Health Insurance Database 2000 (LHID 2000), a subset of the NHIRD. The LHID 2000 dataset contains outpatient and inpatient care data for one million randomly sampled beneficiaries enrolled in the NHI system in 2000. There were no significant differences in the distributions of age, sex, and healthcare costs between the individuals in the LHID and the NHIRD [19]. Since the dataset was released for research purposes and the patients included in the dataset had been anonymized, the study was exempt from the need for written informed consent from the subjects. The study was approved by the Institutional Review Board of Fu-Jen Catholic University (FJU-IRB No: C104014).

### 2.2. Study Design and Study Population

**Figure 1:**
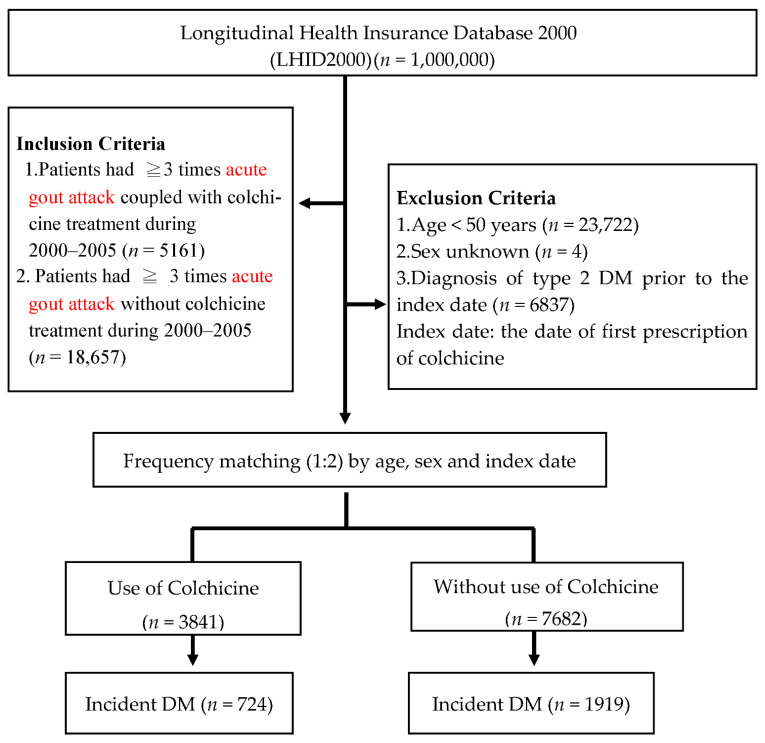
Flowchart of the study sample selection. DM, diabetes mellitus.

The present study was based on patients newly diagnosed with gout during an outpatient or inpatient visit between 1 January 2000, and 31 December 2005. To ensure the diagnostic validity of gout, we identified gouty patients as fulfilling the following criteria: three or more clinical visits with a primary diagnosis of gout with documented uric acid therapy. Gouty patients who received colchicine for more than 90 days (equal to three prescriptions in outpatient visits between 1 January 2000, and 31 December 2005) were defined as the exposed cohort. In comparison, gouty patients who did not undergo colchicine treatment were considered the unexposed cohort. The date of the first prescription of colchicine was designated as the index date. For each gouty patient treated with colchicine (the exposed cohort), two (1:2) gouty patients who did not receive colchicine (the unexposed cohort) were frequency-matched by age, sex, and index date. Cohort members were excluded from the study if they were aged <50 years (*n* = 23,722), had been diagnosed with T2DM prior to the index date (*n* = 6837), or had unknown demographics (*n* = 4). Finally, we enrolled a total of 11,523 subjects, including 3841 gouty patients with colchicine treatment and 7682 gouty patients without colchicine treatment. Follow-up began on the index date and continued until the first occurrence of the following: T2DM (outcome of interest), death (as assessed by disenrollment from NHI), or the end of follow-up on 31 December 2013, whichever occurred first (Figure 1).

### 2.3. Ascertainment of Type 2 Diabetes Mellitus

The primary outcome of interest was the occurrence of new-onset T2DM. To identify patients with T2DM with sufficient accuracy, we determined patients with at least three outpatient diagnoses of T2DM and excluded the diagnoses of type 1 DM.

### 2.4. Covariate Assessment and Adjustment

In the present study, patient demographics, including age, sex, comorbidities, and co-medication prescriptions, were identified as covariates. We used inpatient and outpatient files to ascertain whether cohort members had comorbidities during the study period, including hypertension, hyperlipidemia, ischemic heart disease, heart failure, chronic liver disease, chronic kidney disease, chronic obstructive pulmonary disease (COPD), and non-alcoholic fatty liver disease (NAFLD). Comorbidities were determined for diagnosed patients with at least three outpatient claims or one inpatient claim during the study period. Co-medication prescriptions were determined using the Anatomic Therapeutic Chemical (ATC) classification system, including non-steroidal anti-inflammatory drugs (NSAIDs), corticosteroids, allopurinol, benzbromarone, and sulfinpyrazone. Evidently, some study participants had comorbidities such as hypertension, ischemic heart disease, and heart failure. Those participants may exhibit consumption of diuretics, beta-blockers, calcium channel blockers, and statins. Therefore, those medication prescriptions were also included in the context of co-medications. Given that data on cigarette smoking and obesity were not available in the NHIRD, smoking-related diseases such as COPD and obesity-associated diseases such as hypertension, hyperlipidemia, and NAFLD were used as substitute measures of smoking and obesity. In the analysis setting, patient demographics, the aforementioned comorbidities, and co-medication prescriptions were included in the regression models for adjustment.

### 2.5. Statistical Analysis

Chi-square and *t*-tests were used to evaluate the differences in distributions of categorical and continuous variables between the study cohorts. In addition, multivariable Cox proportional hazard regression models were performed to compute hazard ratios (HRs) with 95% confidence intervals (CIs) to examine the association of colchicine treatment with the risk of T2DM after adjusting for potential confounders. A log-minus-log plot of survival was used to verify that the study variables analyzed satisfied the proportionality assumption of the Cox regression model [20]. All statistical tests were two-sided, and a level of 0.05 was considered statistically significant. All data analyses were conducted using SAS software, version 9.1 (SAS Institute, Cary, NC, USA).

## 3. Results

The characteristics of the study cohorts are shown in Table 1. There were no significant differences in the distributions of age and sex between the study cohorts due to the matching scheme. In general, significant differences were identified in the distributions of comorbidities between study cohorts. In addition, more patients receiving colchicine were undergoing treatments with allopurinol, benzbromarone, sulfinpyrazone, diuretics, beta-blockers, calcium channel blockers, and statins, but fewer of those patients were users of NSAIDs and corticosteroids compared with patients in the comparison cohort.

During the follow-up period, there were 724 and 1919 incident T2DM cases in the colchicine and non-colchicine cohorts, respectively. The cumulative incidence of T2DM was significantly lower in the colchicine-user cohort (18.8%) than in the comparison cohort (25.0%). Results on the association between colchicine use and T2DM risk are presented in Table 2. After adjusting for potential confounders, the risk of T2DM was significantly lower in the colchicine-user cohort than in the comparison cohort (adjusted HR, 0.74; 95% CI, 0.36–0.87).

The stratified analyses based on subgroups formed by sex and age showed results consistent with the primary findings, namely that an inverse association between colchicine use and the risk of T2DM was evident in both sexes and different age groups. Adjusted HRs (95% CIs) were 0.72 (0.32–1.18) and 0.81 (0.23–0.96) for males and females, respectively, as well as 0.76 (0.23–1.12) and 0.82 (0.31–0.92) for those aged 50–64 years and ≥65 years, respectively (Table 3).

## 4. Discussion

This retrospective cohort study based on nationwide claims data from Taiwan’s NHIRD demonstrated that gouty patients using colchicine had a reduced risk of T2DM compared with those not using colchicine. Moreover, this inverse association between colchicine use and diabetic risk was evident in both sexes and different age groups.

It has been noted that hyperglycemia increases markers of inflammation and the enhancement of oxidative stress [21,22]. Oxidative stress and chronic inflammatory process induce the development of insulin resistance, leading to the development of diabetes [23,24]. Thus, inflammation plays an important role in the pathogenesis of T2DM. Therefore, anti-inflammatory medications may ameliorate diabetes, preventing its progression and vascular complications. More importantly, prospective studies have indicated that diabetic patients have a two- to four-fold risk of developing coronary artery disease and myocardial infarction [25], establishing that T2DM is an independent risk factor for cardiovascular disease (CVD) [25,26,27]. Indeed, there is evidence that the interaction of T2DM and related cardiovascular risk underpins the progressive nature of the vascular damage, leading to atherosclerosis [25]. Therefore, the management of T2DM is crucial for better care for individuals at risk of developing CVDs.

Colchicine is an alkaloid of *Colchicum autumnale* L. with a specific anti-inflammatory effect in gout attack [8]. Colchicine can bind the tubulin molecule, thereby inhibiting its polymerization into microtubules in vitro [28]. In many inflammatory conditions, neutrophils are the pivotal cells involved. The participation of neutrophils in inflammation depends upon their ability to migrate towards the damaged tissue [28]. Since neutrophil migration is affected by microtubules, the disruption of microtubules in neutrophils by colchicine to inhibit their migration can suppress the inflammatory process [8,29]. There is evidence that reactive oxygen species (ROS) play an important role in the pathogenesis of many inflammatory diseases [30]. For colchicine, a radical scavenging/antioxidant effect was suggested. Animal studies showed that colchicine reduces the inflammatory response and enhances the antioxidant capacity in rats fed with a high-fat diet [31]. In addition, colchicine treatment induced a protective effect on oxidative stress, the antioxidant redox system, and Ca2 released in serum and polymorphonuclear leucocytes of familial Mediterranean fever patients in remission that are characterized by oxidative stress [32]. Thus, colchicine treatment could induce a protective effect on diabetic risk via a regulatory effect on inflammatory processes and oxidative stress.

Indeed, a decreasing trend of developing diabetes associated with increasing duration of colchicine exposure was documented in research conducted on veterans in the United States [33]. Furthermore, a randomized controlled trial identified that colchicine significantly improved obesity-associated inflammatory variables and showed an improvement in fasting insulin resistance, with trends toward improving fasting glucose, fasting insulin, and insulin-independent glucose disposal [34]. In agreement with previous findings, this study provides longitudinal evidence that colchicine treatment in gouty patients was associated with a decreased risk of T2DM. As both micro and macrovascular complications of diabetes are closely associated with inflammation, with its anti-inflammation properties, colchicine might reduce the risk of micro and macrovascular complications of diabetes.

The major strength of this study is that it used a nationwide dataset from the NHIRD of Taiwan, which allowed analyses to be conducted in a real-life setting in an unselected patient population. Furthermore, for our cohort study, there was the advantage that the data were collected from a population-based dataset with prospectively documented and virtually completed data on drug prescriptions, thus minimizing the potential disadvantages of selection bias and information bias. However, the methodological limitations of this study need to be noted. It has been noted that research conducted on the basis of insurance claims datasets is often prone to bias because the information on confounders contained in an insurance dataset is generally limited [35]. Therefore, some potential confounders that are linked to diabetic risk, including smoking habits, dietary patterns, physical activity, and obesity, were not available in the NHIRD. Although we used surrogate measures of smoking status and obesity as potential confounders for adjustment, we cannot exclude the possibility that the link between colchicine exposure and diabetic risk might have contributed to residual confounding due to the fact that surrogate variables may not capture all of the risks. Additionally, claims data in the NHIRD lack laboratory parameters; thus, laboratory data such as blood sugar, glycated hemoglobin, and uric acid levels, could not be integrated into the analysis. Further, we could not verify the uric acid condition in individuals with gout following colchicine administration. Moreover, the information on patient compliance to colchicine treatment was not taken into consideration in our analyses. Finally, we defined gouty patients who received colchicine for more than 90 days as the exposure cohort. Whether 90 days is sufficient exposure to colchicine given the long-term follow-up is an open question.

## 5. Conclusions

In conclusion, our nationwide population-based cohort study provides longitudinal evidence that the use of colchicine in gouty patients was associated with a reduced risk of T2DM. This inverse relationship between colchicine treatment and diabetic risk was consistently found across each set of subgroup analyses. Of note, we should be cautious in interpretating our findings given the lack of body mass index and other T2DM risk data in the NHIRD. Further studies are needed to determine the clinical implications of the present study.

## Figures and Tables

**Table 1:** Baseline characteristics of study cohorts.

Variable	Without Colchicine (*n* = 7682)	With Colchicine (*n* = 3841)	*p* Value
Age (years)			1.000
50–59	2432 (31.7%)	1216 (31.7%)	
60–69	2340 (30.5%)	1170 (30.5%)	
≥70	2910 (37.9%)	1455 (37.9%)	
Sex			
Female	1168 (15.2%)	584 (15.2%)	1.000
Male	6514 (84.8%)	3257 (84.8%)	
Comorbidities			
Heart failure	572 (7.4%)	387 (10.1%)	<0.001
Chronic liver disease	1633 (21.3%)	713 (18.6%)	<0.001
Chronic kidney disease	539 (7.0%)	573 (14.9%)	<0.001
Hyperlipidemia	1789 (23.3%)	787 (20.5%)	0.001
Hypertension	4993 (65.0%)	2639 (68.7%)	<0.001
Ischemic heart disease	2475 (32.2%)	1289 (33.6%)	0.148
NAFLD	214 (2.8%)	106 (2.8%)	0.936
COPD	2076 (27.0%)	1030 (26.8%)	0.812
Co-medications			
NSAIDs	4742 (61.7%)	1892 (49.3%)	<0.001
Corticosteroids	1353 (17.6%)	516 (13.4%)	<0.001
Allopurinol	503 (6.5%)	646 (16.8%)	<0.001
Benzbromarone	780 (10.2%)	686 (17.9%)	<0.001
Sulfinpyrazone	60 (0.8%)	54 (1.4%)	0.001
Diuretics	2901 (37.8%)	1836 (47.8%)	<0.001
Beta-blockers	4455 (58.0%)	2409 (62.7%)	<0.001
Calcium channel blockers	2382 (31.0%)	1464 (38.1%)	<0.001
Statins	2346 (30.5%)	1334 (34.7%)	<0.001

NAFLD, non-alcoholic fatty live disease; COPD, chronic obstructive pulmonary disease; NSAIDs, non-steroidal anti-inflammatory drugs.

**Table 2:** Association between colchicine administration and risk of type 2 diabetes mellitus.

Variable	No. of Subjects	No. of DM Cases	Cumulative Incidence (%)	Adjusted HR (95% CI)
Overall				
Without Colchicine	7682	1919	25.0	1.00
With Colchicine	3841	724	18.8	0.74 (0.36–0.87)

Note: DM, diabetes mellitus; HR, hazard ratio; CI, confidence interval. Hazard ratios were adjusted for age; sex; index date; comorbidities, including hypertension, hyperlipidemia, heart failure, ischemic heart disease, chronic liver disease, chronic kidney disease, non-alcoholic fatty liver disease, and chronic obstructive pulmonary disease; and use of concomitant medications, including non-steroidal anti-inflammatory drugs, corticosteroids, allopurinol, benzbromarone, sulfinpyrazone, diuretics, beta blockers, calcium channel blockers, and statins.

**Table 3:** Association between colchicine administration and risk of type 2 diabetes mellitus stratified by sex and age.

Variable	No. of Subjects	No. of DM Cases	Cumulative Incidence (%)	Adjusted HR (95% CI)
Gender				
Males				
Without colchicine	6514	1535	23.6	1.00
With colchicine	3257	564	17.3	0.72 (0.32–1.18)
Females				
Without colchicine	1168	384	32.9	1.00
With colchicine	584	160	27.4	0.81 (0.23–0.96)
Age				
50–64				
Without colchicine	3581	1069	29.9	1.00
With colchicine	1789	374	20.9	0.76 (0.23–1.12)
≥65				
Without colchicine	4101	850	20.7	1.00
With colchicine	2052	350	17.0	0.82 (0.31–0.92)

Note: DM, diabetes mellitus; HR, hazard ratio; CI, confidence interval. Hazard ratios were adjusted for age; sex; index date; comorbidities, including hypertension, hyperlipidemia, heart failure, ischemic heart disease, chronic liver disease, chronic kidney disease, non-alcoholic fatty liver disease, and chronic obstructive pulmonary disease; and use of concomitant medications, including non-steroidal anti-inflammatory drugs, corticosteroids, allopurinol, benzbromarone, sulfinpyrazone, diuretics, beta blockers, calcium channel blockers, and statins.

## Data Availability

No additional data are available.
